# Changes in socio‐economic inequality in alcohol‐attributable mortality in periods of increasing and decreasing alcohol affordability

**DOI:** 10.1111/dar.13989

**Published:** 2024-12-12

**Authors:** Pia Mäkelä, Elsi Lindell

**Affiliations:** ^1^ Finnish Institute for Health and Welfare Helsinki Finland

**Keywords:** alcohol, alcohol affordability, income, mortality, socio‐economic factors

## Abstract

**Introduction:**

Reducing alcohol affordability reduces alcohol‐related harm but its impact on socio‐economic inequalities requires further study. We examine changes in alcohol‐attributable mortality inequalities in Finland during periods of sharply rising (2000–2007) and falling (2008–2017) alcohol affordability.

**Methods:**

Linking individual‐level register data on causes of death and socio‐demographics for the Finnish population aged ≥25 in 2000–2017 (68 million person‐years), we analysed age‐standardised monthly alcohol‐attributable mortality rates by sex and income quintile (*n* = 32,699 alcohol‐attributable deaths). Regression models were used to analyse mortality trends in the two periods, contrasting high‐ and low‐income groups.

**Results:**

Inequalities in alcohol‐attributable mortality between low‐ and high‐income groups were large throughout the study period. During the period of rising alcohol affordability, mortality increased among high‐income men with an average monthly increase of 0.17% (*p* = 0.046). This rate was even higher among low‐income men, increasing by 0.55% per month, that is, +0.38 percentage points more than the rate for high‐income men (*p* = 0.002). Among women, mortality increased at similar rates in both income groups. During the period of falling alcohol affordability, mortality decreased among high‐income men with an average monthly decrease of −0.21% (*p* < 0.001), and it decreased even more among low‐income men (−0.40%, i.e., −0.19 percentage points more, *p* = 0.030). Among women, the decreases were not statistically significant.

**Discussion and Conclusions:**

The results indicate that increased alcohol affordability was associated with widening socio‐economic inequalities while reduced affordability was linked with narrowing inequalities among men. Reducing alcohol affordability is thus a recommendable policy for reducing socio‐economic inequality in alcohol‐related harm.

**Key Points**

- Inequalities in alcohol‐attributable mortality between income groups were large in Finland.
- When alcohol affordability rose, alcohol‐attributable mortality increased in all income groups, and among men in the low‐income group in particular.
- When alcohol affordability was reduced, alcohol‐attributable mortality decreased among men in both low‐ and high‐income groups, but more in the low‐income group.
- Reducing alcohol affordability through alcohol taxes, minimum unit pricing or other means seems to be an effective way to reduce socio‐economic inequalities in severe alcohol‐attributable harm.

## 1 INTRODUCTION

Alcohol use increases the risk of a wide range of adverse health and social outcomes and significantly reduces life expectancy in many countries [1, 2]. This burden from alcohol use is not evenly distributed among individuals. Poor and vulnerable individuals, or more generally people with a low socio‐economic status in society, suffer from alcohol‐attributable harm much more than those who are better off [3, 4]. This is true for all‐cause mortality as well, but the socio‐economic inequalities for alcohol‐attributable mortality are 1.5–2 times greater than those for all‐cause mortality [3]. In some European countries such as Denmark, Finland, Hungary and Slovenia, alcohol‐attributable conditions play an important role in generating inequalities in life expectancy [4].

These inequalities go against the UN Sustainable Development Goals [5], the central principle of which is ‘Leave no one behind’, and especially against Goal 10, which is expressly about reducing inequality within and among countries. Similarly, the World Health Organization European Programme of Work calls for ‘action to address the persistent health inequalities in and between countries across WHO Europe's work’ [6]. There is good will to improve the situation. However, evidence on effective methods to tackle inequities in alcohol‐related harm is scarce [7].

Despite the limited evidence on effective methods to address socio‐economic inequalities in alcohol‐attributable harm, there is some support for the effectiveness of policies that increase the price of alcohol [7, 8]. Modelling studies have indicated that volumetric taxation and minimum unit pricing have the greatest potential to reduce socio‐economic inequality in alcohol‐attributable harm [9, 10]. The introduction of minimum unit pricing was indeed followed by reduced socio‐economic inequalities in alcohol‐related hospitalisations and mortality in Scotland [11]. Finnish evidence from the 2004 alcohol tax cuts showed that lowering alcohol taxes had the most impact on alcohol‐related mortality and hospitalisations in lower socio‐economic groups [12, 13]. For policymakers aiming to reduce socio‐economic inequality in alcohol‐related mortality, the critical question is whether the impact is symmetrical, meaning that an increase in alcohol taxes or reduced alcohol affordability would also reduce these inequalities. The addictive properties of alcohol can cast doubt on this symmetry assumption.

In Finland, the 2000s have seen large changes in alcohol affordability. In the early 2000s, alcohol affordability first strongly rose, as excise taxes on alcohol were cut by one‐third, on average, in 2004 and the economy expanded. Alcohol taxes were cut because tourist import quotas on alcohol from the EU were abolished and Estonia, a neighbouring country of Finland with cheap alcohol, joined the EU [14]. Affordability then strongly fell in 2008–2017, as alcohol taxes were moderately raised five times (in 2008, twice in 2009, in 2012, and in 2014) and the economy temporarily slumped. The alcohol tax in euros reached the pre‐2004 level in 2009 for wine, in 2012 for beer, in 2021 for spirits and in 2014 for the consumption‐weighted average of beverages (vm.fi/alkoholiverotus). According to statistics on causes of death [15], alcohol‐attributable deaths strongly increased in 2000–2007 and then strongly decreased in 2007–2017. This created a favourable setting for establishing how such society‐level reductions and increases in alcohol taxes, or alcohol affordability more generally, are reflected in socio‐economic inequality in alcohol‐attributable mortality.

Hence, our aim is to examine what happened to socio‐economic inequalities in alcohol‐attributable mortality in Finland, first during a period of rising alcohol affordability in 2000–2007, and then during a period of falling alcohol affordability in 2008–2017. In 2018, a new alcohol act came into force, and for this reason the year 2017 was chosen as an ending point for the study period.

## 2 METHODS

### 2.1 Data

The study population consisted of Finnish residents aged 25 or older in 2000–2017. Younger people were excluded from the study because income levels are more comparable in a population with completed formal education. We defined the study population using the FOLK Basic register data module maintained by Statistics Finland [16]. The module includes annual individual‐level socio‐demographic information on all individuals with Finnish permanent residence as of 31 December each year.

As a measure of socio‐economic status, we used income quintiles. We defined income for each individual as the total disposable household income divided by the number of household consumption units measured using the modified Organisation for Economic Co‐operation and Development equivalence scale [17]. For people who did not belong to a household dwelling unit (2.7%, on average), this was undetermined and personal disposable income, when available, was used instead. We determined income quintiles annually for each individual according to the income distribution of the Finnish population aged 25 years or older. Each year, on average 0.4% of the study population could not be linked with any income information and were merged with the lowest income quintile.

Using identification numbers unique to each individual, we linked the data with Statistics Finland's Causes of Death register data. Deaths were considered alcohol‐attributable if the underlying cause of death was a wholly alcohol‐attributable International Classification of Diseases, Tenth Revision, code (alcohol‐related disorders F10, alcoholic cardiomyopathy I42.6, alcoholic liver disease K70, alcohol‐induced chronic or acute pancreatitis K86.0 or K85.2, accidental alcohol poisoning X45 or one of the less common code categories G31.2, G40.51, G62.1, G72.1, K29.2, O35.4, P04.3, Q86.0).

### 2.2 Statistical method

For descriptive analyses, we calculated annual age‐standardised mortality rates per 100,000 person years in each income quintile for men and women using the direct method and the age structure of the overall study population in 2017 as the reference.

To further assess income‐related inequalities in the temporal changes of alcohol‐attributable mortality, we analysed monthly mortality rates by income group using segmented regression models. The low‐income group was defined as the lowest income quintile. The high‐income group consisted of the two highest income quintiles since the number of alcohol‐attributable deaths was insufficient in the highest earning 20% alone to permit robust statistical inference. Because the study population was defined at the end of each year, month‐to‐month changes in the population structure could not be directly observed. To approximate these by income, sex and age, we used log‐linear interpolation. Using this data, we calculated age‐standardised monthly mortality rates by gender and income group.

Prior to conducting the regression analysis, we examined seasonal patterns in income‐group‐specific time series describing alcohol‐attributable mortality. The Kruskal‐Wallis test comparing monthly means in alcohol mortality indicated seasonality in the series of the low‐income men, while for high‐income men and for both income groups among women the test indicated no statistically significant evidence of seasonality. We obtained seasonally adjusted series for low‐income men using the so called TRAMO/SEATS procedure, specifically the R package RJDemetra [18], an R implementation of the Eurostat program for seasonal adjustment JDemetra+.

The model equation can be written as follows.

$$\ln\left(Y_{t}\right)=\beta_{0}+\beta_{1}t+\beta_{2}X+\beta_{3}\mathrm{Xt}+\beta_{4}I_{t}+\beta_{5}tI_{t}+\beta_{6}XI_{t}+\beta_{7}\mathrm{Xt}I_{t}+\varepsilon_{t.}$$

*Y*_*t*_ is the age‐standardised mortality rate observed in each month *t* = 1, …, 216, where *t* = 1 corresponds to January 2000 and *t* = 216 corresponds to December 2017. The models were fitted for the natural logarithm of the dependent variable using ordinary least squares regression which allows the exponentiated parameter estimates to be interpreted as relative rates (RR) compared to the reference, with (RR − 1) × 100% yielding estimated percentage differences. Time t is included as an explanatory variable to model average monthly change (trend) in mortality. Variable *X* indicates the income group, with the high‐income group taken as the reference.

The variable *I*_*t*_ describes the time period or segment in the models. As suggested by the literature and confirmed in the preliminary analysis of the income‐group‐specific mortality rates, two temporal segments could be distinguished within the study period. The variable *I*_*t*_ is therefore defined as a binary indicator with *I*_*t*_ = 1 after December 2007 and *I*_*t*_ = 0 otherwise. Defined this way, the parameters β_0_, β_1_, β_2_, and β_3_ describe the average development of mortality in the first period of rising alcohol affordability, while the corresponding parameters β_4_, β_5_, β_6_, and β_7_ indicate the shift in these parameters for the period with decreasing alcohol availability relative to the first period. This model specification allows the comparison of the two income groups as well as the shifts in the slope and level of mortality to be estimated directly; estimates for the development of the inequalities in the second period are obtained using linear combinations of parameters (e.g., the sum of β_1_ and β_5_—or the product of corresponding RRs—is a measure for the slope in the high‐income group in the second period). Appendix 1 includes an explanation of how the equation translates to each of the income–time segment combinations.

As a sensitivity analysis, we assessed the development of mortality not attributable to alcohol to see whether the observed pattern was specific to alcohol‐attributable deaths.

The analyses were conducted using the R programming language version 3.6.6 [19] in Statistics Finland's FIONA remote access system.

## 3 RESULTS

In the 18 years the study population was followed, the data covered 68 million person‐years, 0.9 million deaths overall, of which 32,699 were alcohol attributable (Table 1). As the quintiles were formed for the whole population by household income, for men the higher‐earning quintiles were somewhat larger than the lower‐earning quintiles, and for women vice versa. Approximately half of the alcohol‐attributable deaths were observed in the lowest income quintile. The fact that other deaths were also more common in these categories suggests that people may drift towards lower incomes for reasons connected to mortality (e.g., ageing, sickness).

**TABLE 1:** Person years and number of alcohol‐attributable and all‐cause deaths in 2000–2017 and mean age by income quintile and gender.

Quintile	Person years	Alcohol‐attributable deaths	All deaths	Age in 2007
Men				
1 = Lowest	5,873,862	12,945	152,263	52.2
2	6,122,185	5434	145,336	55.8
3	6,598,513	3239	71,841	49.9
4	6,980,727	2216	41,881	47.9
5 = Highest	7,148,567	1726	30,809	49.3
All men	32,723,853	25,560	442,130	50.9
Women				
1 = Lowest	7,758,515	3522	228,521	60.3
2	7,347,032	1545	131,660	57.8
3	6,956,056	984	47,469	50.9
4	6,617,334	631	24,497	48.7
5 = Highest	6,505,797	457	16,684	49.3
All women	35,184,733	7139	448,831	53.8
All	67,908,586	32,699	890,961	52.3

**FIGURE 1:**
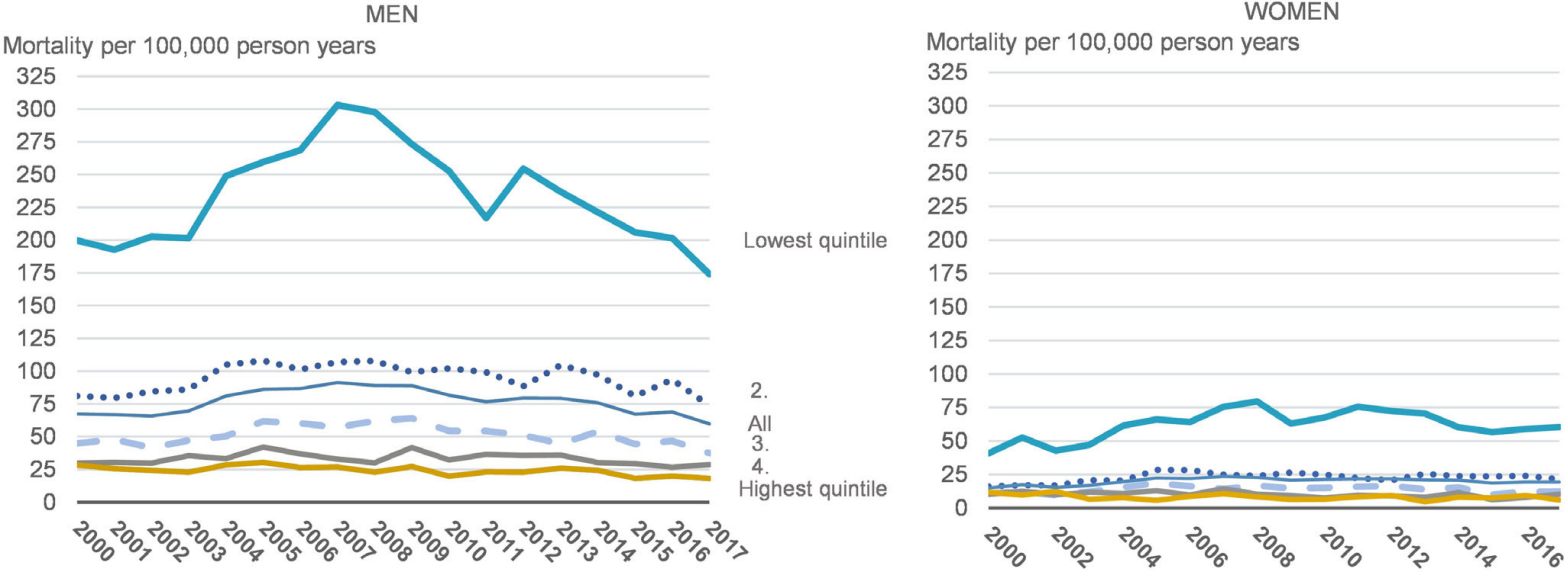
Alcohol‐attributable mortality per 100,000 person‐years by income quintile and gender in 2000–2017.

There was a strong gradient in alcohol‐attributable mortality across income groups among both men and women (Figure 1). The difference between the lowest and the second lowest income group was particularly large.

Between 2000 and 2007, when alcohol‐related mortality increased, this change was particularly striking in the lowest income group in both genders (Figure 1). However, when the shift in the trend occurred, the favourable reduction in alcohol‐attributable mortality also benefited the poorest income group the most. This was particularly the case among men, for whom the rate of alcohol‐attributable mortality in the lowest income group declined to the early 2000s level towards the end of our study period, while among women the rates in the lower income groups remained at a somewhat higher level than before the alcohol tax reduction in 2004.

**FIGURE 2:**
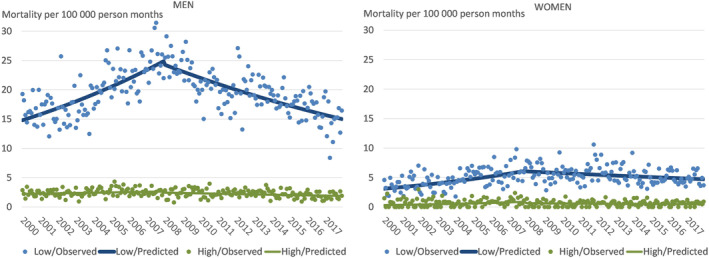
Model‐fitted alcohol‐attributable mortality (solid line) and the observed alcohol‐attributable mortality (dots) per 100,000 person‐months for the lowest income quintile and the two highest income quintiles combined, by gender (men on the left, women on the right) 2000–2017 (observed values based on three or fewer observations were censored by Statistics Finland for data security reasons when exporting data [not in modelling]: These were replaced by zeros in the graph [almost all cases appeared among women in the high‐income group, in which ca. one in four observations were missing for this reason]).

The results from regression models for alcohol‐attributable mortality and its changes in the lowest income group compared to the two highest income groups are depicted in Table 2 in terms of parameter estimates, in Figure 2 as fitted values, and the translation of the parameter estimates to rates of monthly change (%) and absolute change during the period are shown in Table 3. The large inequalities between the socio‐economic groups were confirmed by the model: at the beginning of the study period, low‐income men had a 6.59‐fold mortality rate compared to high‐income men (RR for β_2_, *p* < 0.001), and for women the corresponding rate ratio was 9.53 (*p* < 0.001). By the beginning of the second period, men's mortality in the low‐income group was 11.84‐fold compared to high‐income men according to the model (product of RRs for β_2_ and β_6_), and 8.77 among women.

**TABLE 2:** Estimates from the model for alcohol‐attributable mortality for men and women, covering years 2000–2007 (period 1, rising alcohol affordability) and 2008–2017 (period 2, falling alcohol affordability).

	Men			Women		
	RR	95% CI	*p*‐value	RR	95% CI	*p*‐value
Period 1						
High‐income group						
β1: trend (monthly change)	1.0017	1.0000, 1.0034	0.046	1.0107	1.0018, 1.0197	0.018
Low‐income group						
β2: Level difference between income groups	6.59	5.79, 7.51	<0.001	9.53	4.79, 18.94	<0.001
β3: Trend difference between income groups	1.0038	1.0014, 1.0061	0.002	0.9965	0.9841, 1.0091	0.584
Period 2						
High‐income group						
β4: Level difference compared to period 1	1.34	1.09, 1.66	0.007	2.58	0.84, 7.89	0.097
β5: Trend difference compared to period 1	0.9961	0.9941, 0.9982	<0.001	0.9866	0.9759, 0.9974	0.015
Low‐income group						
β6: Level difference between income groups compared to period 1	1.80	1.33, 2.43	<0.001	0.92	0.19, 4.48	0.918
β7: Trend difference between income groups compared to period 1	0.9944	0.9915, 0.9973	<0.001	1.0043	0.9890, 1.0198	0.585

Abbreviations: CI, confidence interval; RR, relative rates.

**TABLE 3:** Point estimates for the monthly rate of change (%) and for the absolute change (mortality per 100,000 person‐years) between the first and last month of the period, by period, income and gender, as derived from the model parameter estimates from Table 2.

	Monthly rate of change, %		Absolute change	
	Men	Women	Men	Women
Period of rising alcohol affordability 2000–2007				
High‐income	0.17	1.07	0.4	0.4
Low‐income	0.55	0.72	10.1	3.0
Period of falling alcohol affordability 2008–2017				
High‐income	−0.21	−0.29	−0.8	−0.3
Low‐income	−0.40	−0.21	−9.9	−1.4

In the period of rising alcohol affordability, alcohol‐attributable mortality increased among high‐income men (Table 2, β_1_: RR = 1.0017, *p* = 0.046) and women (RR = 1.0107, *p* = 0.018), indicating an average monthly increase of 0.17% and 1.07%, respectively. However, among men alcohol‐related mortality increased more rapidly in the low‐income group compared to the high‐income group (β_3_: RR = 1.0038 compared to the high‐income group, *p* = 0.002). This translates to a 0.38 percentage points greater increase than in the high‐income group, or a monthly increase of altogether 0.55%. Among women, the difference between income groups in the rate of increase in alcohol‐attributable mortality was not statistically significant (β_3_: RR = 0.9965, *p* = 0.584; point estimate for women's low‐income group: altogether a 0.72% increase monthly).

In absolute terms, the contrast between the income groups in the estimated increase in mortality was even larger, because even the same relative increase means many more deaths when the starting level is high: according to the predicted values shown in Figure 2, among men the relative change from the first to the last month of the period 2000–2007 was 68% in the low‐ and 18% in the high‐income group while the absolute increase was +10.1 and +0.4 deaths per 100,000 person‐months, respectively (Table 3), that is, in the low‐income group the absolute increase was 25 times larger compared to the high‐income group. For women, among whom the relative changes were similar in the two income groups, the absolute changes were +3.0 and +0.4, that is, the absolute increase was 6.9 times larger among low‐income women.

The estimates for the second period—when alcohol affordability fell—show that the upward trend of the first period saw a statistically significant downward shift in 2007 for both men's and women's high‐income group (Table 2, β_5_, *p* < 0.001 and *p* = 0.015, respectively) and an even stronger downward shift for men's low‐income group (β_7_, *p* < 0.001). In the second period, among high‐income men alcohol‐attributable mortality decreased (RR = 0.9979, as indicated by the combination of parameter estimates for β_1_ and β_5_, *p* < 0.001) implying an average monthly decrease of 0.21% (see also Table 3). Among low‐income men, alcohol‐attributable mortality decreased more rapidly than in the high‐income group (RR = 0.9981 compared to the high‐income group, *p* = 0.030; as indicated by the combination of parameter estimates for β_3_ and β_7_), that is, the average monthly decrease was 0.19 percentage points greater than in the high‐income group, or altogether 0.40% monthly. Among higher income women, the decrease in the second period was not statistically significant (RR = 0.9971, or a 0.29% monthly decrease, *p* = 0.374), and the difference between income groups in the rate of decrease was not statistically significant, either (RR = 1.0008, *p* = 0.863; point estimate for the decrease in this group was altogether a monthly decrease of 0.21%).

In absolute terms, when mortality decreased, the model indicates a greater improvement in the low‐income group (Table 3): among men the decrease was −9.2 deaths per 100,000 person‐months in the low‐income group and −0.6 in the high‐income group (a 17‐fold difference), and −1.3 and −0.2 among women (a 7.2‐fold difference).

We examined whether this pattern of first increasing and then decreasing mortality was specific to alcohol‐attributable deaths. For deaths not attributable to alcohol, the mortality rates mostly decreased throughout the period (Appendix 2).

## 4 DISCUSSION

This study corroborated the existence of very significant socio‐economic inequalities in alcohol‐attributable mortality. It further demonstrated that during a period of rising alcohol affordability in Finland in 2000–2007, among men the increase in alcohol‐attributable mortality was stronger in the low‐income group, that is, the socio‐economic inequalities widened. Among women, the relative increase was similar for low‐ and high‐income groups. In the period of falling alcohol affordability in 2008–2017, the decrease in alcohol‐attributable mortality was stronger in the low‐income group among men, that is, socio‐economic inequalities narrowed, while among women the relative inequality did not change.

The increase in alcohol‐attributable mortality in Finland following the alcohol tax cuts in 2004 has been previously documented [14], along with the finding that the increase in alcohol‐attributable mortality in early 2000s was greater in lower socio‐economic groups [12, 20]. Tarkiainen et al. [21] have also reported that the reduction in alcohol‐attributable mortality contributed to reducing socio‐economic inequalities in life expectancy between 2006–2009 and 2011–2014. We tested whether this finding of widened inequalities in alcohol‐attributable mortality following rising alcohol affordability is symmetrical, that is, whether also falling affordability would be followed by reduced levels and inequalities in alcohol‐attributable mortality despite the addictive properties of alcohol. The Finnish case in 2007–2017 strongly suggests that the effect works both ways, at least among men. Among men, both the increase and the decrease in alcohol‐attributable mortality were stronger in the low‐income group even in relative terms, and as the base level is much higher in that group, in absolute terms the changes in the low‐income group were vastly greater (a 25 times greater increase and a 17 times greater decrease). The finding of a greater decrease in alcohol‐attributable mortality among lower‐income men when alcohol affordability fell supports the conclusions from previous modelling studies of Meier et al. [10] who found that four different alcohol taxation and pricing policies were expected to lead to reductions in health inequalities, and this was particularly so with minimum unit pricing (see also [9]). Findings from Scotland showed that the actual introduction of minimum unit pricing reduced alcohol‐attributable mortality the most in the 40% most socio‐economically deprived areas [11].

Among women, the findings depended on whether socio‐economic inequality was measured by relative or absolute differences. Alcohol‐attributable mortality among women increased in the first period with rising alcohol affordability but the relative inequality did not; in the second period with falling affordability neither the decreasing trend nor the relative difference in it between the income groups were statistically significant. Because of the great inequalities in the base levels, however, the absolute changes were greater in the lower socio‐economic group (7 times greater increase in the first period and a 7 times greater decrease in the second, compared to the high‐income group). A partial explanation for the smaller impacts among women could be that both the levels of alcohol‐attributable mortality and its inequalities were somewhat less pronounced among them than they were for men.

There may be two separate but intertwined reasons for the greater benefit of reduced alcohol affordability for low‐income groups. First, the price elasticity of alcohol is greater among poorer individuals than among the more well‐off [22]. Those with less money need to prioritise their consumer choices more, and alcohol is likely to be among the non‐essential goods for most. However, the higher impact of prices on alcohol‐related harm in low‐income subgroups is not solely due to reduced alcohol consumption. According to a systematic review by Probst et al. [3], only 27% of the socio‐economic inequalities in alcohol‐related mortality could be explained by alcohol use, mostly heavy episodic drinking. This finding is known as the alcohol harm paradox: the same amounts of alcohol cause disproportionate damage in poorer individuals and communities compared to affluent ones [23]. The reasons for the alcohol harm paradox are not entirely clear but may include interactions with other health behaviours, differential access to and quality of health care, and varying levels of social support [24]. These factors can magnify both the damage caused by increased alcohol use and the benefits of reduced alcohol use in disadvantaged populations.

The strong connection between income and alcohol‐attributable mortality results from a reciprocal impact: it is partly due to the effects of poverty on alcohol‐attributable mortality and partly due to the impacts of problem drinking on incomes (e.g., due to loss of work or health problems; [25, 26]). Regardless of the direction of the causal arrow, the strongest observed effects of changes in alcohol affordability on alcohol‐attributable mortality have been seen in the most disadvantaged part of the population, which has suffered from the highest rates of alcohol‐attributable mortality throughout the period, while observed impacts in higher‐income groups have been much smaller. However, this may not be the whole truth. Herttua et al. [12] showed that in the 2 years following the 2004 tax cuts, in absolute terms alcohol‐attributable mortality increased most among groups with the highest levels of alcohol‐attributable mortality such as the unemployed, early‐age pensioners, and low‐income groups. However, a 4‐year follow‐up of alcohol‐attributable hospitalisations [13] found the impact to be more even. Therefore, it cannot be concluded that higher‐income groups are largely unaffected by price changes. Among them, the road from policy changes to alcohol‐related mortality may be longer and first involve reduced incomes due to deteriorating health or unemployment. Confirmation of this hypothesis requires further study.

A limitation of our study design is that it cannot prove causality. In the relatively long periods examined, other societal changes occurred besides changes in alcohol affordability. Most other factors are unlikely to have changed course in 2007, but the national economic situation did change over the two periods [27]. This change is part of the changing affordability: when people have more disposable income, some of it is used to buy alcohol, and vice versa [28], and this effect is expected to be stronger in low‐income groups [22]. However, the economy may also have broader impacts beyond alcohol affordability. For example, hypothetically, the accessibility of healthcare could change with the national economic situation. However, such general factors would be expected to affect mortality from all causes, but the pattern of changes was shown to be specific to alcohol‐related mortality. No control group was available that would be otherwise similar but unaffected by the changes in alcohol affordability, so some uncertainty remains. Nevertheless, the fact that the tax policy was first strongly changed in one direction, then in the other, and in both cases the socio‐economic inequalities at least among men changed accordingly, improves our confidence that the connection between changes in alcohol affordability and changes in alcohol‐attributable mortality is likely to be causal.

In conclusion, the Finnish evidence from the 2000s strengthens the evidence base that reducing alcohol affordability is a good tool to reduce socio‐economic inequality in severe alcohol‐attributable harm. Reviews and editorials about solutions to reduce socio‐economic inequality in alcohol‐attributable harm [3, 29, 30, 31] have stressed the importance of comprehensive strategies, addressing alcohol as part of a network of factors contributing to inequalities. This means addressing socio‐economic inequalities at different levels and stages, starting with root causes such as tackling insufficient education and poverty, and incorporating efficient alcohol policy tools such as taxation and availability. Commercial and political determinants [32] must also be addressed to change social environments to support healthy choices.

## AUTHOR CONTRIBUTIONS

Each author certifies that their contribution to this work meets the standards of the International Committee of Medical Journal Editors. ChatGPT was used to check the English language written by the non‐native authors.

## CONFLICT OF INTEREST STATEMENT

None.

## Data Availability

Access to the data supporting the findings of this study can be applied for from Statistics Finland. Access is subject to fees (see https://stat.fi/tup/tutkijapalvelut/index_en.html).

## APPENDIX 1: HOW DOES THE MODEL EQUATION TRANSLATE TO DIFFERENT SUBGROUPS AND COMPARISONS?

The model equation was:

$$\ln\left(Y_{t}\right)=\beta_{0}+\beta_{1}t+\beta_{2}X+\beta_{3}\mathrm{Xt}+\beta_{4}I_{t}+\beta_{5}tI_{t}+\beta_{6}XI_{t}+\beta_{7}\mathrm{Xt}I_{t}+\varepsilon_{t}$$

where

- $Y_{t}$ is the age‐standardised mortality rate observed in month *t*.
- t is a continuous variable indicating time measured in months and t=1,…,216. Defined this way, t=1 corresponds to January 2000 and t=216 corresponds to December 2017.
- X is an indicator variable for income group, with the high‐income group as the reference.
- $I_{t}$ is an indicator variable marking the change in the time segments after December 2007, with $I_{t}=1$ for t>96, and $I_{t}=0$ otherwise.

The first four parameters describe the model in the first time period, and the following four parameters indicate the difference in these parameters in the second period relative to the first period. In our setting, the first period covered the years 2000–2007 and the second the years 2008–2017.

Below we show how the model translates to each time segment and income group.

First segment, high‐income group:

$$\ln\left(Y_{t}\right)=\beta_{0}+\beta_{1}t+\varepsilon_{t}$$

Second segment, high‐income group:

$$\ln\left(Y_{t}\right)=\beta_{0}+\beta_{1}t+\beta_{4}+\beta_{5}t+\varepsilon_{t}$$

$$\ln\left(Y_{t}\right)=\left(\beta_{0}+\beta_{4}\right)+\left(\beta_{1}+\beta_{5}\right)t+\varepsilon_{t}$$

That is, $\beta_{1}+\beta_{5}$ indicates the slope (increase or decrease in time) in mortality of the high‐income group during the second segment of time.

First segment, lower income:

$$\ln\left(Y_{t}\right)=\beta_{0}+\beta_{1}t+\beta_{2}+\beta_{3}t+\varepsilon_{t}$$

$$\ln\left(Y_{t}\right)=\left(\beta_{0}+\beta_{2}\right)+\left(\beta_{1}+\beta_{3}\right)t+\varepsilon_{t}$$

That is, $\beta_{1}+\beta_{3}$ indicates the slope (increase or decrease in time) in mortality of the low‐income group, and $\beta_{3}$ indicates the difference in slopes between income groups.

Second segment, lower income:

$$\ln\left(Y_{t}\right)=\beta_{0}+\beta_{1}t+\beta_{2}+\beta_{3}t+\beta_{4}+\beta_{5}t+\beta_{6}+\beta_{7}t+\varepsilon_{t}$$

$$\ln\left(Y_{t}\right)=\left(\beta_{0}+\beta_{2}+\beta_{4}+\beta_{6}\right)+\left(\beta_{1}+\beta_{3}+\beta_{5}+\beta_{7}\right)t+\varepsilon_{t}$$

That is, $\beta_{1}+\beta_{3}+\beta_{5}+\beta_{7}$ indicates the slope for the low‐income group, and $\beta_{3}+\beta_{7}$ can be interpreted as the difference in steepness of the lower income group's monthly trend compared to the high‐income group.
